# Anti-Inflammatory and Anti-asthmatic Effects of TMDCT Decoction in Eosinophilic Asthma Through Treg/Th17 Balance

**DOI:** 10.3389/fphar.2022.819728

**Published:** 2022-02-08

**Authors:** Yumei Zhou, Haihong Zhao, Tieshan Wang, Xiaoshan Zhao, Ji Wang, Qi Wang

**Affiliations:** ^1^ National Institute of TCM Constitution and Preventive Medicine, School of Chinese Medicine, Beijing University of Chinese Medicine, Beijing, China; ^2^ Beijing Research Institute of Chinese Medicine, Beijing University of Chinese Medicine, Beijing, China

**Keywords:** Tuo-Min-Ding-Chuan decoction, Treg/Th17, eosinophilic asthma, anti-inflammatory, anti-asthmatic

## Abstract

Tuo-Min-Ding-Chuan decoction (TMDCT) is a Traditional Chinese Medicine (TCM) formula consisting of twelve herbs that can relieve the symptoms and treat allergic asthma. Yet, the underlying mechanism of action is still unclear. In this study, we investigated the effect of TMDCT in regulating Treg/Th17 cells immune balance and explored potential metabolic and gut biomarkers associated with Treg and Th17 cells in eosinophilic asthma mice treated by TMDCT. We found that TMDCT increases Treg cells percentage and decreases Th17 cells percentage in the ovalbumin (OVA) -induced eosinophilic asthma mice model. Furthermore, Imidazoleacetic acid, dL-glutamine, L-pyroglutamic acid, 2-deoxy-d-glucose were preliminary identified as biomarkers in plasma metabolites treated by TMDCT, meanwhile genus *Desulfovibrio*, genus *Butyricimonas* and genus *Prevotella 9* were preliminary identified as gut microbiota biomarkers after TMDCT treatment. These results provide an experimental foundation for the treatment of allergic asthma with Chinese herbal compounds.

## Introduction

Asthma is a chronic inflammatory disease which is difficult to control affects more than 300 million people worldwide. With current rising trends, it is expected that this number will reach 400 million people by 2025. According to some reports, there are nearly 250,000 asthma-related deaths each year, many of which are avoidable (Christiansen and Zuraw, 2019).

Inflammatory disorder in allergic asthma is characterized by broncho-construction, bronchial hyper-responsiveness, and even tissue damage and the most common type of asthma (National Asthma Education and Prevention Program, 2007). If not properly treated, allergic asthma can progress to chronic obstructive lung disease or other disease associated with airways and lung tissue. The burden of allergic diseases has increased over recent years, as evidenced by a high incidence of patients suffering from these diseases and incurring high financial costs (Bousquet et al., 2016). Over the years, anti-inflammatory drugs have been developed, such as inhaled steroids and bronchodilators that can relieve symptoms. Yet, existing drugs cannot completely cure the patient, and have an elevated recurrence rate. Moreover, conventional drugs have been associated with certain side effects. E.g., an inhaled corticosteroid (ICS), the dominant treatment in type 2-high asthmatic inflammation, can suppress the endocrine system, which assists body’s immune system fight against infection, while long-term steroid treatments can lead to anxiety and depression.

Allergic asthma is induced by an abnormal type 2 immune response to inhaled allergens, such as house dust mites (HDM), grass pollen, animal dander, and mold (Caminati et al., 2018). In the airway, the inflammatory response is mainly caused by Th2 type inflammation, inducing cytokines IL-4, IL-5, and IL-13 overexpression, which consequently activate the expression of IgE and the infiltration of eosinophils and mast cells in the airway.

Previous studies have shown that the imbalance of Th1/Th2 cells may be involved in the pathogenesis of airway inflammation in asthma (Berker et al., 2017). Besides that, some recent studies suggested that insufficient Th1 cell differentiation is not the only cause leading to over-differentiation and activation of Th2 cells. It has been discovered that Treg cells have an essential role in regulating the body’s immune balance. Dysfunctional Treg cells, unable to effectively suppress excessive Th2 response, may lead to asthma and other allergic diseases.

Th2-type inflammation is involved in all types of asthma (mild, moderate, and severe). Also, inflammation caused by Th17 cells has been associated with a progression of asthma (Israel and Reddel, 2017). The imbalance between Treg cells and Th cells such as Th1, Th2 and Th17 cell responses in allergic asthma is associated with the development of allergic asthma (Noval Rivas and Chatila, 2016). Genetic and immunological evidence suggested that Treg cells can promote tolerance to allergens and prevent allergic disorders (Palomares et al., 2014). Thus, it is believed that the inflammation of allergic asthma inflammation and increasing Treg cells number or stimulating the proliferation of immunosuppressive cells such as Treg cells is a new strategy for the treatment of allergic asthma.

Recently, some new drugs with the ability to exert Treg cells in the body’s immune system or stimulate differentiation of Treg cells have been tested. Although great development has been achieved using allergen immunotherapy (AIT) and other immunotherapy, treatment of allergic asthma and its recurrence remains challenging. New treatment options that can regulate the immune balance of Treg/Th17 are currently emerging.

TMDCT is a TCM formula consisting of twelve herbs that can relieve the symptoms and treat allergic asthma. Nevertheless, the underlying mechanism of action is still unclear. TMDCT contain 12 kinds of traditional Chinese medicine, it can suppresses inflammation of allergic asthma and inhibit the degranulation of mast cell (Qin et al., 2021). In our study, we investigated the effect of TMDCT in regulating Treg/Th17 cells balance and explored potential metabolic and gut biomarkers associated with Treg and Th17 cells in eosinophilic asthma mice treated with TMDCT using non-targeted metabolome and 16S rDNA technology.

## Materials and Methods

### Mice

BALB/c mice (female, 6–8 weeks old, 17–20 g) were obtained from Beijing Vital River Laboratory Animal Technology Co., Ltd in China. Keeping mice in specific pathogen-free conditions in Beijing University of Chinese Medicine. All mice were kept at a controlled room (25 ± 1°C, 45–60% humidity). All animal studies were conducted in accordance with the institutional animal care regulations of Beijing University of Chinese Medicine and were conducted in accordance with AAALAC and IACUC guidelines.

### OVA-Induced Allergic Asthma Mice Model and Grouping

**FIGURE 1:**
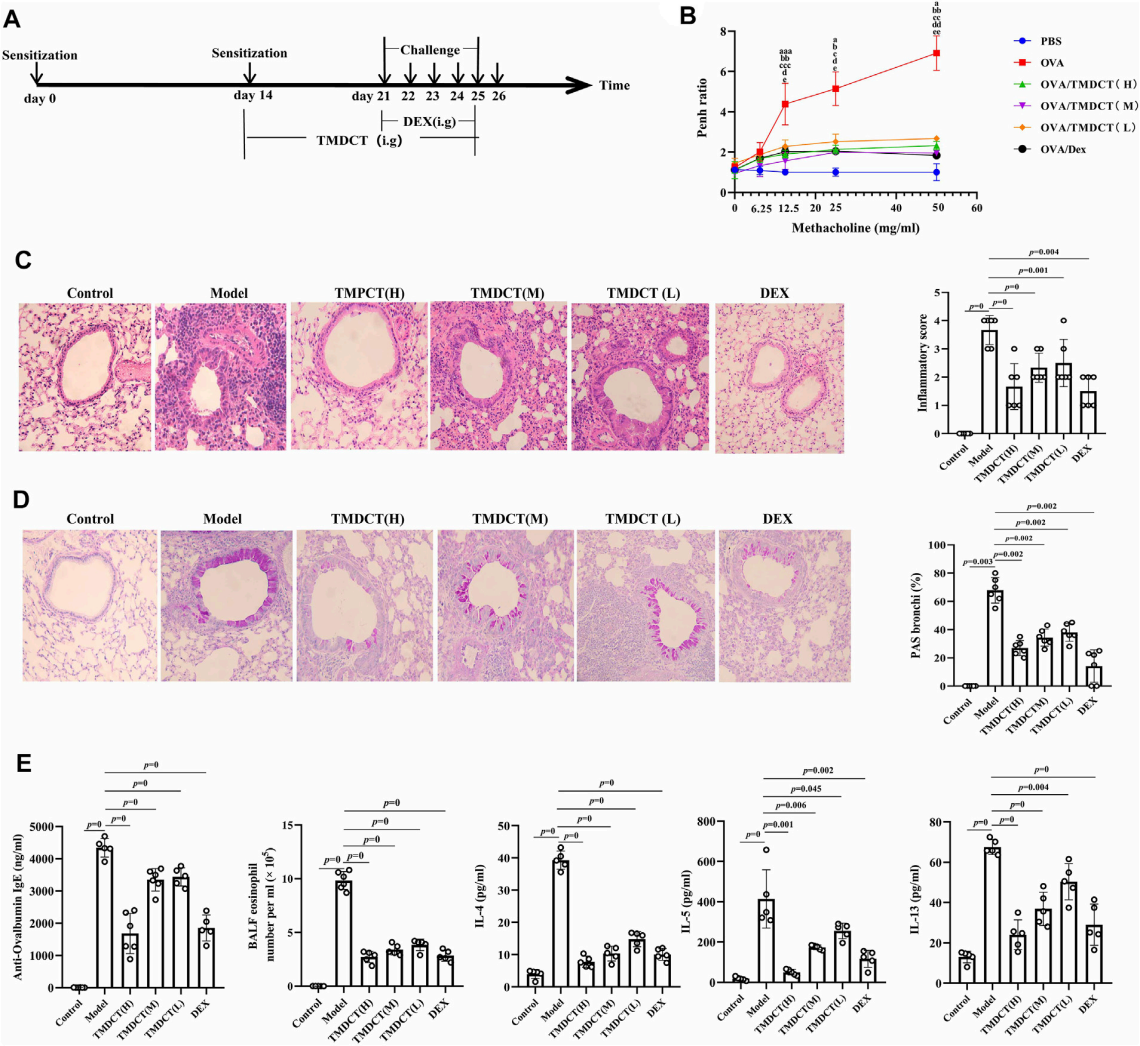
TMDCT can alleviate allergic inflammation in eosinophilic asthma BALB/c mice. **(A)** Experimental protocol for eosinophilic asthma induced by OVA and treatment administration. **(B)** Lung function experiments. Detection the Penh value when the methacholine concentration is 0, 6.25, 12.5, 25, 50 mg/ml respectively. Penh ratio (average Penh in the 5-minute interval with methacholine divided by the average Penh in the 5-minute interval with PBS). a, *p* < 0.05 vs. Control; aa, *p* < 0.01 vs. Control; aaa, *p* ≤ 0.001 vs. Control. b, *p* < 0.05 vs. TMDCT(H); bb, *p* < 0.01 vs. TMDCT(H); bbb, *p* ≤ 0.001 vs. TMDCT(H). c, *p* < 0.05 vs. TMDCT(M); cc, *p* < 0.01 vs. TMDCT(M); ccc, *p* ≤ 0.001 vs. TMDCT(M). d, *p* < 0.05 vs. TMDCT(L); dd, *p* < 0.01 vs. TMDCT(L); ddd, *p* ≤ 0.001 vs. TMDCT(L). **(C)** Left: HE staining of lung tissues; Right: Inflammatory score results. **(D)** Left: PAS staining of lung tissues; Right: Total lung inflammation was defined as the average of the peribronchial and perivascular inflammation scores. **(E)** Detection if specific OVA-IgE in serum; eosinophils count in BALF; detection of cytokines IL-4, IL-5, IL-13. All the values are expressed as mean +/- SEM. n = 4-5 animals per group.

The OVA-induced eosinophilic asthma BALB/c mice model was constructed following a previously described approach (Dumas et al., 2018). Briefly, mice were intraperitoneally injected with 2 mg of OVA (Sigma-Aldrich, Cat#A5503) mixed with 2 mg Imject™ Alum Adjuvant (Invitrogen, Cat#77161) and PBS on day 0 and day 14. From the 21st to 25th days post-injection, in the challenge phase, mice are continuously nebulized with 1% OVA for 30 min as shown in Figure 1A.

OVA-induced allergic asthma mice were then divided in the following groups: High-dose TMDCT group (40.56 g/kg/d TMDCT (H group)), Middle-dose TMDCT treatment group (20.28 g/kg/d TMDCT (M group)), Low-dose TMDCT treatment group (10.14 g/kg/d TMDCT (L group)), and positive group (dexamethasone 1 mg/kg/d). TMDCT, containing 12 kinds of Chinese medicine, was prepared as previously described (Qin et al., 2021). TMDCT and dexamethasone (1 mg/kg/d, a positive drug for suppressing eosinophilic asthma) were given daily at the same time from the 21st to 25th days 1 hour before challenge.

### Detection of Measured Airway Responsiveness

We used 0, 6.25, 12.5, 25, and 50 mg/ml methacholine (mch) (Sigma, Cat#A2251) to detect enhanced pause (Penh), which reflected lung function according to noninvasive measurement of airway hyperresponsiveness by whole-body phelthysmography (WBP-4MR, TOW, China) (Sun et al., 2021).

### Histological Sections and Pathology Scoring

Lungs were fixed with 4% paraformaldehyde (PFA) at 4°C, after paraffin-embedded sections and stained them with hematoxylin and eosin (HE) (to examine cell infiltration detection) or periodic acid-schiff stain (PAS) (to examine mucus production) in lung tissues. Scoring of inflammatory cells and goblet cells was performed in at least three different fields for each lung section as described (Duan et al., 2004). The mean scores were calculated using five animals.

### Bronchoalveolar Lavage Fluid, Serum Cytokines, and the Culture Supernatant of Treg and Th17 Cells Cytokines Analysis

Twenty-four hours after the last aerosol challenge, bronchoalveolar lavage (BAL) fluid was collected by syringe three times with 1 ml PBS containing 1% BSA. Eosinophils in BAL fluid were counted by cell sorting and counting instrument. Cytokine levels of IL-4, IL-5, IL-13, IL-10, IL-17A, IL-6 in BALF were analyzed using a premixed AimPlex™ multiplex-assay kit (Cat#T2C0710709), TGF-β was analyzed using a premixed AimPlex™ multiplex-assay kit (Cat#B111206); OVA-specific IgE in serum was detected by ELISA kit (Cayman, Cat#500840).

### Flow Cytometry Detection of Treg and Th17 Cells in Spleen Tissues

The spleen tissue was aseptically removed and prepared into a single-cell suspension. For Th17 (CD3^+^CD4+IL17A+) cells and Treg cells (CD3^+^CD4^+^ CD25 + FOXP3+) detection, the cells were stimulated by cocktail A for 4 h (BD, Cat# 550583). Samples were then washed and re-suspended in 1 × PBS stained with FVS 780 (BD, Cat#565388) to discriminate viable cells and then incubated with various surface markers. Consequently, samples were fixed by eBioscience Fix/Perm (Cat#00-5523-00) or BD Fix/Perm buffer kit (Cat#554714) to destroy the cell membrane and then were stained with FOXP3 (eBioscience, Cat#17-5773-82), IL-17A (BD, Cat# 564169). In the study, CD3 (BD, Cat# 557,666), CD4 (BD, Cat# 552,775) and CD25 (BD, Cat# 558642) were used. Finally, samples were analyzed with the LSR Fortessa cell analyzer (BD) and BD FACSDiva 8.0.3 software.

### Real-Time PCR Detection of Foxp3 and RORγt mRNA

Foxp3 and RORγt mRNA in the lung tissues were detected. RNA extraction kit (Tiangen Biotech (Beijing) co, LTD, Cat#DP419) was used. RNA reverse transcription into cDNA using a cDNA Synthesis Kit (ThermoFisher, Cat#K1622). The primers sequence was the following: β-actin (FP: GACCCAGATCATGTTTG AGACCT; RP: TCC​AGG​GAG​GAA​GAG​GAT​GC); RORγ (FP: CGCACCAACCT CTTTTCACG; RP: TGG​CAA​ACT​CCA​CCA​CAT​ACT​G); Foxp3 (FP: CTTCAAGT ACCACAATATGCGACC; RP: GCGAACATGCGAGT AAACCAA).

### Untargeted Metabolomics Detection of Plasma

Plasma was collected with 1.5 ml Eppendorf Tubes containing EDTA (ethylene diamine tetraacetic acid), which were centrifuged at 4°C by 1,500 g, 15 min. Rremove the protein by Methanol/acetonitrile (1:1, v/v) and centrifuged at 14000g, 4°C for 15 min. All LC-MS analyses were performed at Shanghai Applied Protein Technology Co., Ltd.

R package (ropls) was used to analyzed the processed data. Unsupervised principal component analysis (PCA) and supervised orthogonal partial least squares discriminant analysis (OPLS-DA) were used to evaluate sample stability. The VIP (variables in the projection) value of the OPLS-DA model was calculated, metabolites with VIP ˃ 1, *p-*value *˂* 0.05 is considered to be the significant changed metabolites.

After multivariate and univariable analysis, searched the significant metabolites in the Human Metabolome database (http://www.hmdb.ca), METLIN (https://metlin. scripps.edu), KEGG (http://www.kegg.com) and Chemspider (http://www. chemspider.com/). Cytoscape software was used to perform enrichment analysis and visualization. Pearson correlation analysis was used to perform and determine the correlation between two variables. KEGG enrichment analysis was performed using MetaboAnalyst (www.metaboanalyst.ca). The volcano plot and clustering analysis were performed using R.

### Gut Microbiota Analysis

Using CTAB/SDS method to extracte the total genome DNA. Then, the V3-V4 regions in 16S rDNA were amplified using a specific primer included in the barcode. Next, an Illumina Miseq/HiSeq2500 platform was used to build a library. We used PCoA (Principal Co-ordinates Analysis) to study the similarity or difference of sample community composition, and LEfSe (LDA Effect Size) to quantify the biomarkers in different groups.

### Statistical Analysis

In this study, *t*-test, one-way ANOVA test (Turkey or Dunnett), Wilcoxon rank-sum test, Kruskal–Wallis test or Wilcoxon rank-sum test was used. *p* value ˂ 0.05 was considered as statistically significant. Pearson analysis with R3.4.2 Heatmap was used to analyze the correlation between metabolites and other index, the correlation between gut microbiota and other index.

## Results

### TMDCT Alleviates Airway Inflammation in BALB/C Mice With Eosinophilic Asthma

To evaluate the treatment effect of TMDCT in treating eosinophilic asthma, an OVA-induced eosinophilic asthma model was constructed as described in Figure 1A. The most significant effect of TMDCT(H) group in reducing cell infiltration (Figure 1C) and decreasing goblet cell hyperplasia was observed in the TMDCT(H) group (Figure 1D)**,** further indicating that TMDCT can alleviate airway inflammation. Moreover, TMDCT reduced airway resistance (Penh ratio), which indicated that lung function in TMDCT with a high concentration group had the best effect (Figure 1B).

To investigate the role of TMDCT in allergic airway inflammation, we detected OVA-specific IgE levels in serum. TMDCT significantly decreased OVA-specific IgE concentration in serum (Figure 1E). Additionally, the eosinophils number in BALF was reduced, and IL-5 levels were decreased in BALF of the TMDCT group, especially in the H group (Figure 1E). Also, TMDCT significantly reduced IL-4 and IL-13 cytokines in BALF. These data suggested that TMDCT could alleviate allergic airway inflammation.

### TMDCT can Increase Treg Cells Percentage and Decrease Th17 Cells Percentage in the Spleen

**FIGURE 2:**
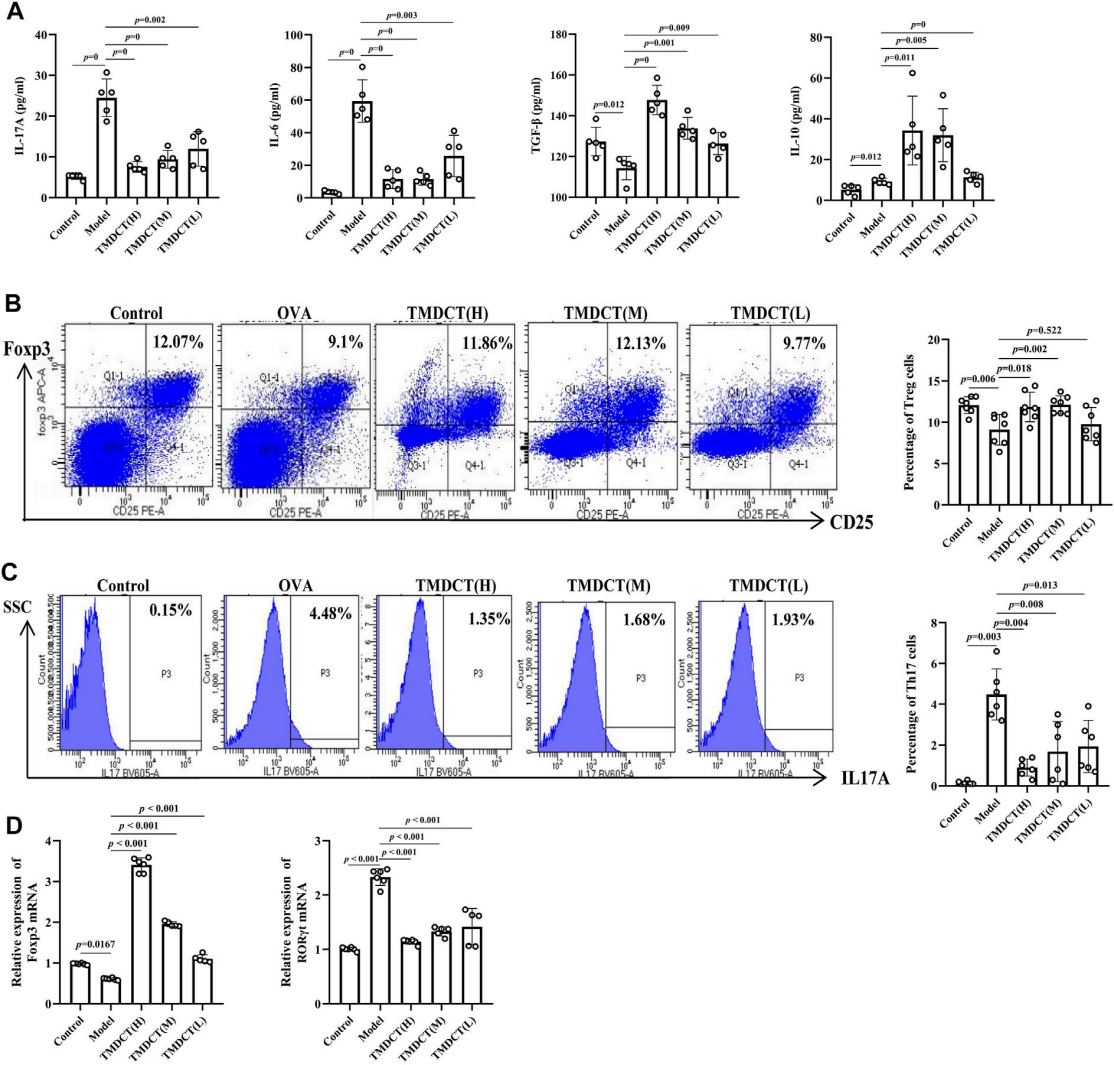
TMDCTregulate Treg/Th17 balance in eosinophilic asthma BALB/c mice. **(A)** Detection of cytokines (IL-17A, IL-6, TGF-β, IL-10). **(B,C)** Left: intracellular staining FOXP3+ and IL-17 in CD4+ T cells in spleen tissue. Right: statistic data of FOXP3+, IL-17+ percentages. **(D)** Real-time PCR data of FOXP3 and RORγt mRNA expression in lung tisue. All the values are expressed as mean +/- SEM. n = 5-7 animals per group.

Th17 and Treg cells have important role in driving and restraining airway inflammation in patients with asthma (Seumois et al., 2020). To further investigate whether TMDCT can regulate immune response, we detected Treg and Th17 cells percentage in spleen tissues. Surprisingly, TMDCT increased Treg cells percentage and decreased Th17 cells percentage; the most significant effect was seen in the TMDCT(H) group (Figures 2B,C).

Next, we analyzed TGFβ, IL-10, IL-6, and IL17A cytokines in BALF. As shown in Figure 2A, TMDCT stimulated TGFβ and IL-10 and suppressed IL-6 and IL17A (*p* value were all <0.05), which is consistent with the Treg and Th17 percentage detection.

At the mRNA level, TMDCT increased the Foxp3 (transcription factor of Treg cells) and decreased RORγt (transcription factor of Th17 cells) relative expression (Figure 2D). These data suggested that TMDCT could decrease Th17 cells percentage, and increase Treg cells percentage in spleen tissues, among them TMDCT(H) has the best effect.

### Identification Plasma Metabolites of TMDCT

**FIGURE 3:**
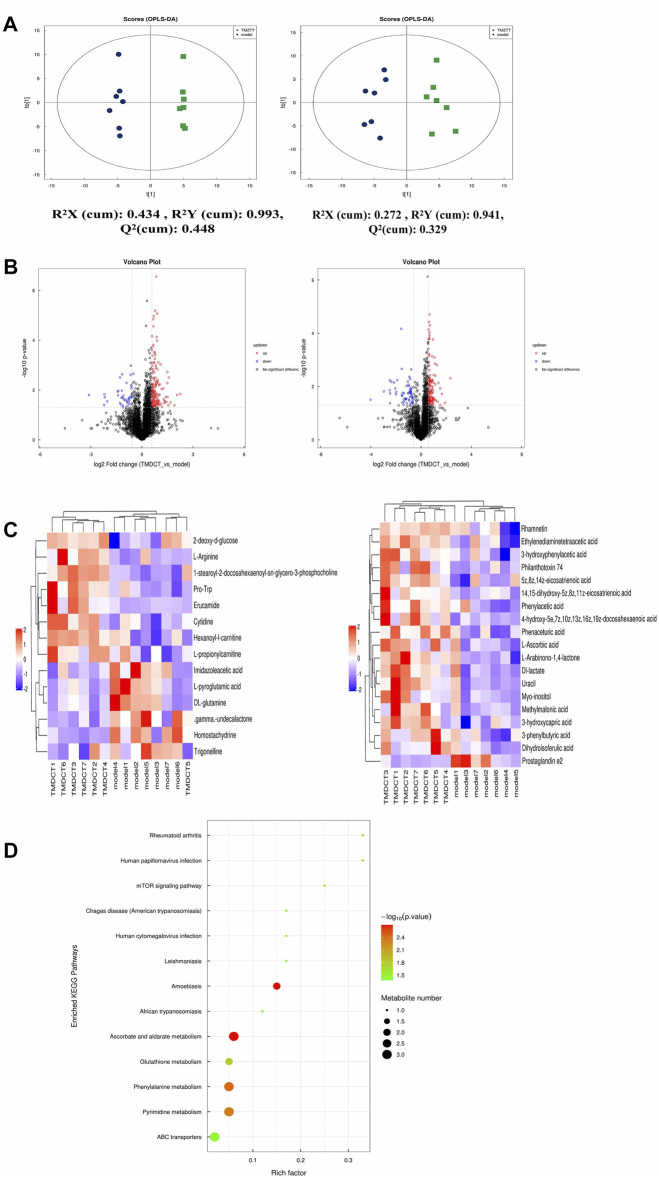
Untarget plasma metabolism detection. **(A)** OPLS-DA analysis of LC/MS data in positive mode (left) and negative mode (right) from TMDCT(H) vs. Model. **(B)** Volcano map of differential metabolites in positive and negative mode. The metabolites with FC > 1.5, *p* value <0.05 are indicated by rose red, satisfying FC < 0.67, *p* value < 0.05 are shown in blue. **(C)** Hierarchical clustering heat map in positive mode (left) and negative mode (right). **(D)** Enriched KEGG pathways based on significant different metabolites between TMDCT(H) group and Model group. n =7 animals per group.

To further explore the mechanism of TMDCT in regulating the immune balance in eosinophilic asthma, we examined potential plasma metabolite biomarkers of TMDCT. The TMDCT with a high concentration was used, and the untargeted metabolomics technology was used in this experiment. As seen in Figure 3A, OPLA-DA analysis found the following: 0.3 < Q2<0.384 (in POS mode) < 0.5, 0.3 < Q2=0.329 (in NEG mode) < 0.5, suggesting that the detected model was stable. The upregulated and downregulated metabolites in positive and negative mode can be shown in Figure 3B, Hierarchical cluster of significant difference metabolites (VIP >1, *p-*value < 0.05) analysis (Figure 3C) further suggested that metabolites in TMDCT or model group had a similar function; they participated in similar metabolite pathway or cell pathway. Finally, six differential metabolites in positive mode and 16 differential metabolites in the negative mode were detected (Table 1)**.**

**TABLE 1:** Differentiated plasma metabolites between model and TMDCT groups.

Name	ESI	VIP	Fold change	*p* Value
1-Stearoyl-2-docosahexaenoyl-sn-glycero-3-phosphocholine	+	8.885118	1.227872	2.63E-06
Cytidine	+	1.081076	1.403976	0.008353
Homostachydrine	+	1.038925	0.655271	0.008604
Pro-Trp	+	1.147062	1.587755	0.012893
Imidazoleacetic acid	+	1.009063	0.890448	0.019436
l-Arginine	+	1.963909	1.903922	0.023805
.gamma.-undecalactone	+	1.286511	0.830919	0.024305
Trigonelline	+	1.412927	0.751799	0.029667
dl-glutamine	+	2.401136	0.898755	0.030164
Hexanoyl-l-carnitine	+	1.586371	1.42347	0.031471
Erucamide	+	7.243441	3.201381	0.031635
l-pyroglutamic acid	+	3.855271	0.90852	0.032281
l-propionylcarnitine	+	2.100705	1.363916	0.03821
2-deoxy-d-glucose	+	1.228756	1.07907	0.039665
Phenaceturic acid	—	1.163085	1.433406	0.004378
L-Arabinono-1,4-lactone	—	1.619284	1.612233	0.005725
4-hydroxy-5e,7z,10z,13z,16z,19z-docosahexaenoic acid	—	1.555208	1.803301	0.006093
Uracil	—	4.134414	1.766824	0.006679
Ethylenediaminetetraacetic acid	—	20.93969	1.229205	0.006782
Philanthotoxin 74	—	1.093587	1.866347	0.010424
3-hydroxycapric acid	—	2.141189	1.513555	0.011763
5z,8z,14z-eicosatrienoic acid	—	1.640482	1.35079	0.013645
3-hydroxyphenylacetic acid	—	4.039256	1.598082	0.014398
Phenylacetic acid	—	2.600282	1.531541	0.016176
dl-lactate	—	7.134456	1.584984	0.018574
Prostaglandin e2	—	3.913766	0.249951	0.019496
l-Ascorbic acid	—	1.732841	1.944992	0.031862
Rhamnetin	—	2.374967	1.098614	0.034419
Myo-inositol	—	1.164396	1.312587	0.037616
14,15-dihydroxy-5z,8z,11z-eicosatrienoic acid	—	1.339304	1.790195	0.041816
Methylmalonic acid	—	1.270319	1.796966	0.04481
Dihydroisoferulic acid	—	1.221032	1.52335	0.047058
3-phenylbutyric acid	—	1.854654	1.270711	0.049813

Next, we performed a KEGG enrichment analysis. As shown in Figure 3D, several pathways were identified. The significant metabolism pathway were our interest.

### Metabolic Biomarkers in Plasma Associated With Treg and Th17 Cells in Eosinophilic Asthma Mice Treated With TMDCT

**FIGURE 4:**
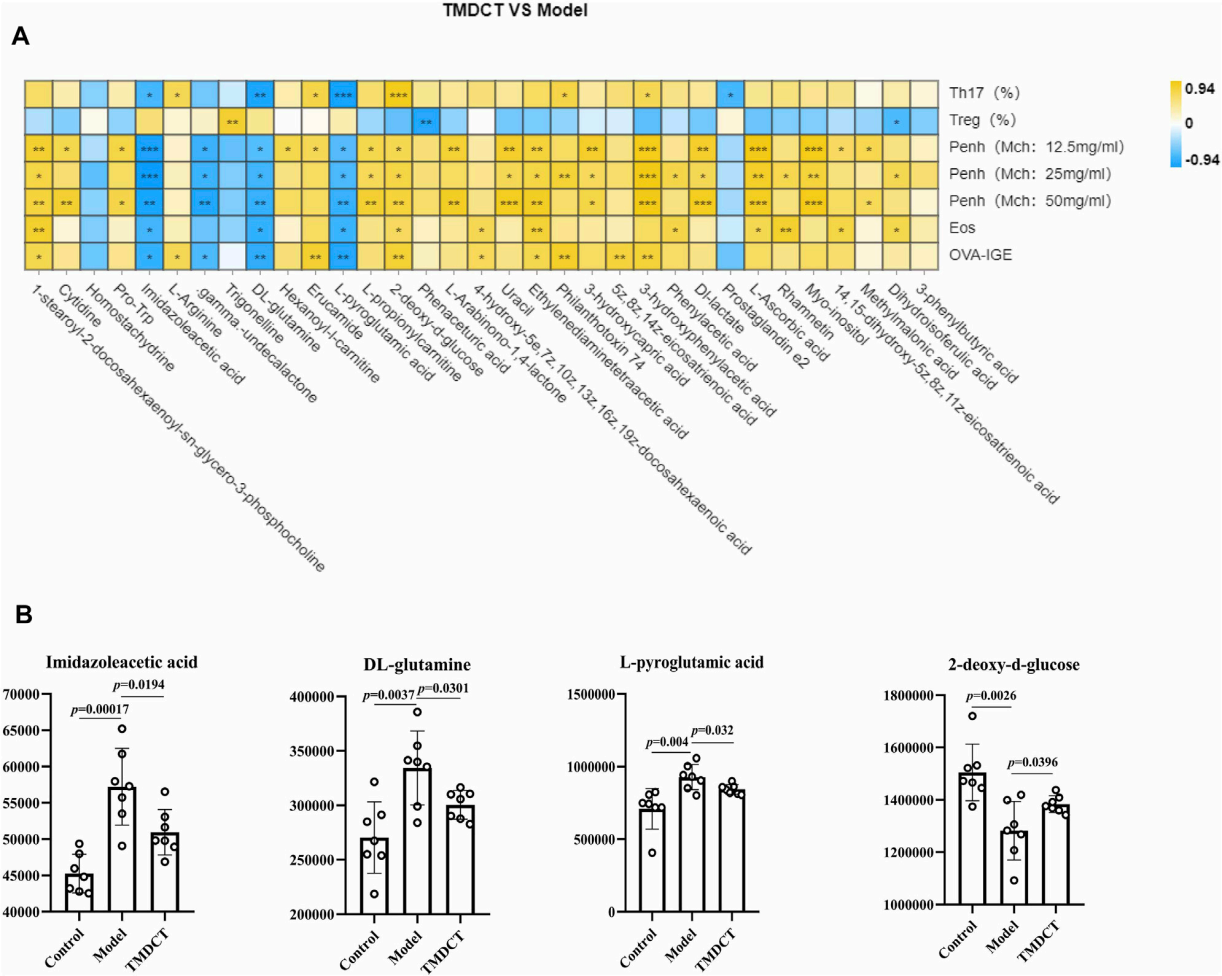
Biomarker selection of plasma metabolites associated with Treg/Th17 balance. **(A)** Correlation analysis of differential plasma metabolites with specific OVA-IgE, Penh value (Mch: 12.5, 25, 50 mg/ml), Eosinophil number in BALF, Treg and Th17 cells percentage.*p < 0.05, **p < 0.01, ***p < 0.001. **(B)** Biomarker in plasma metabolites associated with Treg/Th17 balance. n = 7 animals per group.

To understand the mechanism of TMDCT in regulating Treg/Th17 cells balance, the different metabolites between TMDCT and model group were used for correlation analysis, including Penh value (Mch:12.5, 25, 50 mg/ml), OVA-IgE, Eosinophils, Treg and Th17 cells percentage. In our study, L-Arabinono-1,4-lactone, Myo-inositol, dl-lactate, Uracil and 1-stearoyl-2-docosahexaenoyl-sn-glycero-3-phosphocholine were associated with Penh value (Mch: 12.5, 25, 50 mg/ml), OVA-IgE, Eosinophils, Treg and Th17 cells percentage (Figure 4A). There were obviously different (*p* < 0.05) in 1-stearoyl-2-docosahexaenoyl-sn-glycero-3-phosphocholine and Ethylenediaminetetraacetic acid either between the model and control group, or the model group and the TMDCT group (Figure 4B), they were all associated with OVA-IgE, Penh value (Mch: 12.5 mg/ml, 25 mg/ml, 50 mg/ml) and Eosinophil numbers while they has no correaltion with Treg or Th17 cells percentage (Figure 4A). Moreover, Imidazoleacetic acid, dl-glutamine, l-pyroglutamic acid were all increased in model group compared with control group, while they were all decreased in TMDCT treatment group (*p* value were all <0.05). Besides that, Imidazoleacetic acid, dl-glutamine, l-pyroglutamic acid were all associated with OVA-IgE, Penh value (Mch: 12.5 mg/ml, 25 mg/ml, 50 mg/ml), Eosinophil numbers and Th17 cells percentage (*p* value all <0.05). As shown in Figure 4B, 2-deoxy-d-glucose was decreased in model group when compared with control group (*p* < 0.05), while it increased in TMDCT treatment group (*p* < 0.05), it is also associated with OVA-IgE, Penh value (Mch: 12.5 mg/ml, 25 mg/ml, 50 mg/ml), Eosinophil number and Th17 cells percentage (*p* value all <0.05). The above four metabolites were not correlated with Treg cells, which indicated that they could also affect the Treg/Th17 balance in eosinophilic asthma. The changed metabolites were enriched to phenylalanine metabolism, pyrimidine metabolism, ascorbate and aldarate metabolism pathway, histidine metabolism, glutathione metabolism and metabolic pathways (Figure 3D).

### Variation of Gut Microbiota and Key Phylotypes of Gut Microbiota in Response to OVA-Induced Eosinophilic Asthma Mice Treated by TMDCT

**FIGURE 5:**
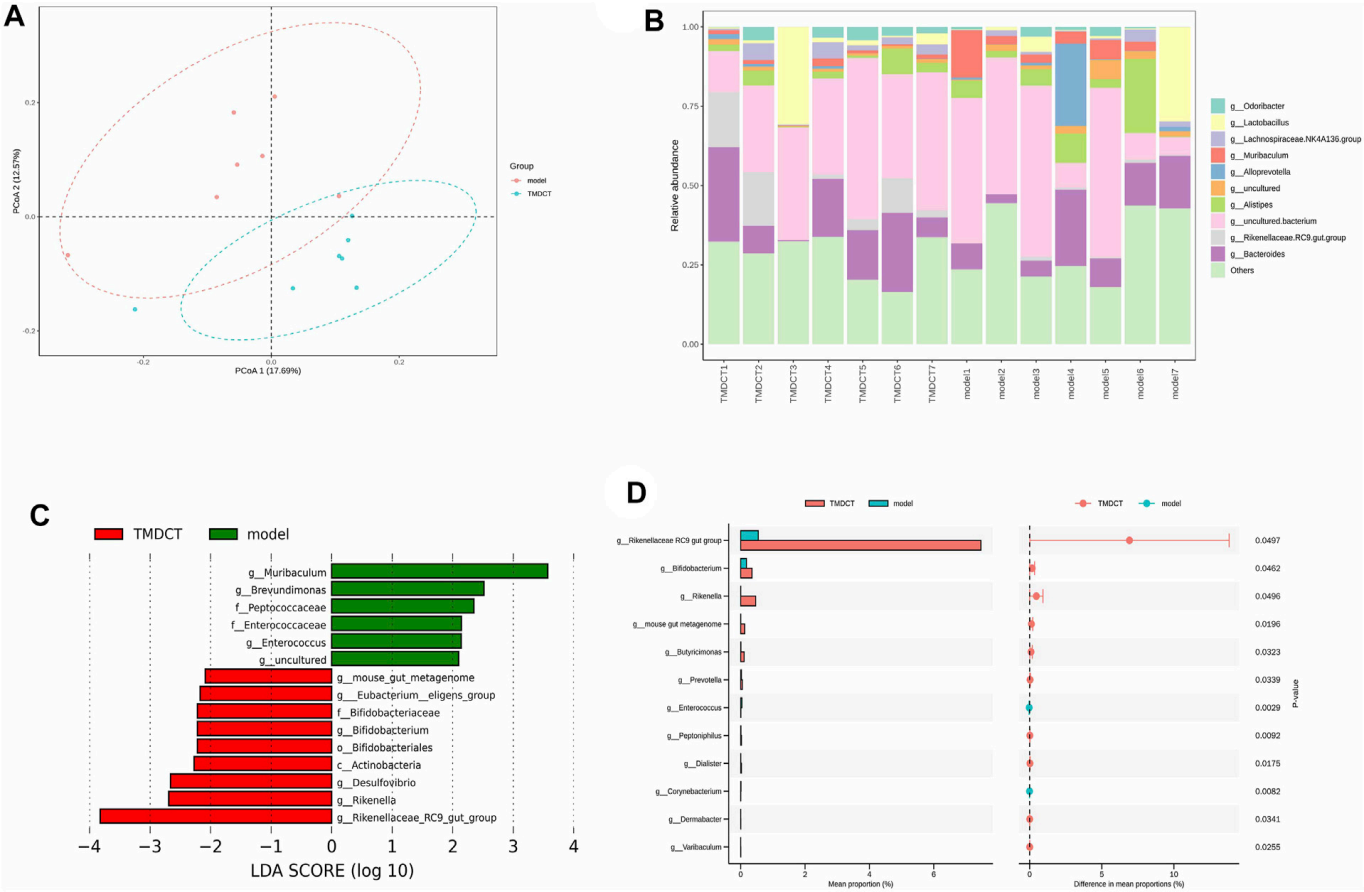
TMDCT significantly changed gut microbiota composition. **(A)** Principal Co-ordinates Analysis (PCoA) analysis in each group. **(B)** Relative abundance of gut microbiota in each group. **(C)** Key phylotypes of gut microbiota in response to TMDCT interventions (LDA method was used). **(D)** Significantly difference gut microbiota analysis, STAMP difference analysis method was used. n = 7 animals per group.

Gut bacteria have an essential role in the action of drugs. Thus, we detected gut microbiota in the Control group and TMDCT (H) group. As shown in Figures 5A,B, the differences in gut microbiota were found between the control and the model group. LEfSe (LDA Effect Size) software was used to discover high-dimensional biomarkers and reveal genome characteristics. LDA ˃ two was used as selection criteria in the study. Through STAMP analysis, we found that genus Rikenellaceae*_ RC9_gut_group* (*p* = 0.0497), genus *Bifidobacterium* (*p* = 0.0462), genus *Rikenella* (*p* = 0.0496), genus *mouse gut metagenome* (*p* = 0.0196), genus *Butyricimonas* (*p* = 0.0323), genus *Prevotella* (*p* = 0.0339), genus *Enterococcus* (*p* = 0.0029), genus *Peptoniphilus* (*p* = 0.0092), genus *Dialister* (*p* = 0.0175), genus *Corynebacterium* (*p* = 0.0082), genus *Dermabacter* (*p* = 0.0341), genus *Varibaculum* (*p* = 0.0255) were different in the TMDCT group compared to the model group in Figures 5C,D.

**FIGURE 6:**
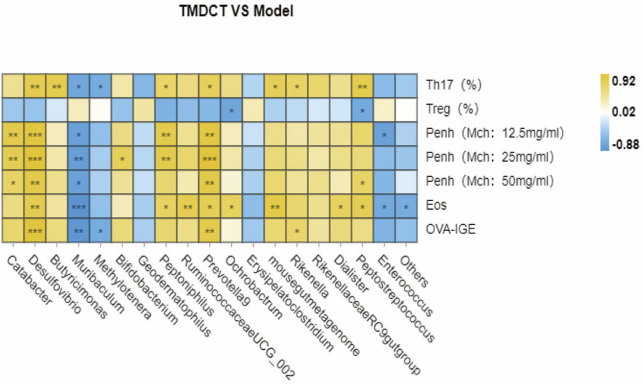
Biomarker selection of gut microbiota associated with Treg/Th17 balance. Correlation of differentiated microbial phylotypes and specific OVA-IgE, Penh value (Mch:12.5, 25, 50 mg/ml), Eosinophil number in BALF, Treg and Th17 cells percentage. *p < 0.05, **p < 0.01, *** *p* < 0.001.

We used Pearson correlation analysis with R 3.4.2 Heatmap software to analyze the correlation. As shown in Figure 6, genus *Desulfovibrio*, genus *Muribaculum* and genus *Prevotella 9* were all associated with Penh value (Mch:12.5, 25, 50 mg/ml), OVA-IgE, Eosinophils number in BALF and Th17 cells percentage. These results indicated a correlation with Treg/Th17 balance in eosinophilic asthma treated by TMDCT.

## Discussion

TMDCT is a prescription of professor Qi Wang used to treat allergic asthma which has significant clinical effect. Yet, its mechanism of action still remains unclear. We further investigated the role of TMDCT in regulating Treg/Th17 cells balance in eosinophilic asthma.

The generation and maintenance of Treg cells and their suppressive cytokines (TGF-β, IL-10) are essential for the induction of allergen tolerance in allergic disease (Palomares et al., 2017). In addition, FOXP3+ Treg cells play a key role in maintaining peripheral tolerance by inhibiting the responsiveness to allergens (Noval Rivas and Chatila, 2016). It is also suggested that allergen-specific immunotherapy regimens increase the numbers of Treg cells (Radulovic et al., 2008). Moreover, some studies found that Treg cells have an irreplaceable role in preventing airway inflammation of allergic asthma (Tortola et al., 2019; Sun et al., 2020). While increasing immunosuppressive function of immunosuppressive cells such as Treg or Breg cells may be essential in a complete cure for allergic asthma.

IL-17A is upregulated in allergic asthma patients, and the upregulation of IL-17A is correlated with the severity of allergic asthma. TGF-β induces the synthesis of the transcription factor of RORγt, which is specific for the Th17 cells (Ivanov et al., 2006). In this study, IL17A and TGF-β, transcription factor RORγt of Th17 cells, and transcription factor FOXP3 of Treg cells were used to analyze the balance of Treg/Th17 cells after TMDCT treatment. Our data suggested that TMDCT can suppress the symptoms of eosinophilic asthma and regulate the balance of Treg/Th17 cells. Interestingly, dexamethasone has similar anti-inflammatory and anti-asthma effects when compared with TMDCT in our study. TMDCT can also treat eosinophilic asthma by regulating Treg/Th17 immune balance the same as dexamethasone does (Kianmehr et al., 2017). While there are too many drawbacks of dexamethasone, a new drug should be explored.

Next, we used non-targeted metabolome to explore potential metabolic biomarkers in the plasma that were associated with Treg and Th17 cells in eosinophilic asthma mice treated with TMDCT. Imidazoleacetic acid, dl-glutamine, 2-deoxy-d-glucose, l-pyroglutamic acid were identified as plasma biomarkers of TMDCT and were all associated with Treg/Th17 cells balance. They were enriched in phenylalanine metabolism, pyrimidine metabolism, ascorbate and aldarate metabolism pathway, histidine metabolism, glutathione metabolism and metabolic pathways, etc. All of the plasma metabolites preliminary proved that TMDCT could regulate Treg/Th17 balance in eosinophilic asthma, which is also the advantage of multi-target effects of traditional Chinese medicine TMDCT. Previous studies found that glutamine can distinct regulate Th17 differentiation (Johnson et al., 2018), which can better explain the reasons for the change in Th17 cells percentage which is consistent with our results. While, until now there are no study found imidazoleacetic acid, 2-deoxy-d-glucose, l-pyroglutamic acid neithor can regulate Treg, Th17 cells nor regulate the differentiation of Treg or Th17 cells, which are not found associated with Treg/Th17 balance in allergic asthma.

Gut, lung, and skin microbiome exposures can also influence the occurrence and the development of allergy disease (Kemter and Nagler, 2019). The gut microbiota is essential in systemic immune regulatory network of allergic disease (Sommer and Bäckhed, 2013) and can regulate drug pharmacokinetics, such as absorption and distribution (Sousa et al., 2008; Tremaroli and Bäckhed, 2012; El Aidy et al., 2015; Zhang et al., 2018). Besides that, the gut-lung axis transfers metabolites and immunomodulatory signals between the gut and lungs. Many studies have shown an increase in the number of respiratory diseases due to deviations in gut ecology (Reddy et al., 2012; Budden et al., 2017). Omics technologies, including metabolomics, can be used to begin to understand relevant molecular changes. In this study, we used omics technology to detect the effect on of TMDCT in regulating Treg/Th17 cells, and then used Non-targeted metabolome and 16S rRNA technology to analyze the biomarkers after TMDCT treatment and relative factors correlated with Treg/Th17 balance. We found that gut microbiota genus Rikenellaceae*_ RC9_gut_group*, genus *Bifidobacterium*, genus *Rikenella*, genus *mouse gut metagenome*, genus *Butyricimonas*, genus *Prevotella*, genus *Enterococcus*, genus *Peptoniphilus*, genus *Dialister*, genus *Corynebacterium*, genus *Dermabacter*, genus *Varibaculum* were significantly different after TMDCT treatment.

*Bifidobacterium* is anti-inflammatory bacteria that can relieve allergic asthma in mice by regulating Th1/Th2 balance (Wang et al., 2020). Allergic patients with chronic asthma usually have low levels of *Bifidobacterium* (Hevia et al., 2016).

Oral administration of *Enterococcus faecalis* can suppress allergic asthmatic response associated with attenuation of Th17 cell development (Zhang et al., 2012). Fecal transplantation containing gut Rikenellaceae *bacteria* can alleviate acute liver injury in mice through regulating Treg/Th17 balance(Liu et al., 2021), while it cannot alleviate eosinophilic asthma. *Corynebacterium* may regulate Th17/Treg cells in the intestinal mucosal immunity (Zhang et al., 2019); However, there are still no studies in allergic asthma. So far, we have not find relevant research about genus *Desulfovibrio*, genus *Muribaculum* or genus *Prevotella 9* are related with Th17 cells percentage in eosinophilic asthma disease, they were the gut microbiota biomarker after TMDCT treatment.

This study has some limitations. First, only the BALB/c eosinophilic asthma model was used in this study. The effect of TMDCT should be further examined in other asthma models. Also, clinical research and other allergic asthma models are required to prove the mechanism of TMDCT in treating allergic asthma. Much more study of the biomarkers of TMDCT treatment should be developed to further explain the mechanism of TMDCT in treating allergic asthma, which will be a new strategy to treat allergic asthma.

## Data Availability

The datasets presented in this study can be found in online repositories. The names of the repository/repositories and accession number(s) can be found below: https://www.ncbi.nlm.nih.gov/, PRJNA799688.
